# Lemon Balm Extracts Prevent Breast Cancer Progression *In Vitro* and *In Ovo* on Chorioallantoic Membrane Assay

**DOI:** 10.1155/2020/6489159

**Published:** 2020-04-14

**Authors:** Roxana Ghiulai, Stefana Avram, Dana Stoian, Ioana Zinuca Pavel, Dorina Coricovac, Camelia Oprean, Laurian Vlase, Claudia Farcas, Marius Mioc, Daliana Minda, Andrei Motoc, Camelia Szuhanek, Corina Danciu, Codruta Soica, Laurentiu Sima

**Affiliations:** ^1^Department of Pharmaceutical Chemistry, Faculty of Pharmacy, Victor Babeş University of Medicine and Pharmacy, 2nd EftimieMurgu Sq., Timişoara 300041, Romania; ^2^Department of Pharmacognosy, Faculty of Pharmacy, Victor Babeş University of Medicine and Pharmacy, 2nd EftimieMurgu Sq., Timişoara 300041, Romania; ^3^Department of Endocrinology, Faculty of Medicine, Victor Babeş University of Medicine and Pharmacy, 2nd EftimieMurgu Sq., Timişoara 300041, Romania; ^4^Department of Toxicology, Faculty of Pharmacy, Victor Babeş University of Medicine and Pharmacy, 2nd EftimieMurgu Sq., Timişoara 300041, Romania; ^5^Department of Environmental and Food Chemistry, Faculty of Pharmacy, Victor Babeş University of Medicine and Pharmacy, 2nd EftimieMurgu Sq., Timişoara 300041, Romania; ^6^Department of Pharmaceutical Technology and Biopharmaceutics, Faculty of Pharmacy, “Iuliu Haţieganu” University of Medicine and Pharmacy, 12 Ion Creangă Street, Cluj-Napoca 400010, Romania; ^7^Department of Pharmaceutical Physics, Faculty of Pharmacy, Victor Babeş University of Medicine and Pharmacy, 2nd EftimieMurgu Sq., Timişoara 300041, Romania; ^8^Department of Anatomy, Faculty of Medicine, Victor Babeş University of Medicine and Pharmacy, 2nd EftimieMurgu Sq., Timişoara 300041, Romania; ^9^Department of Pedodontics and Orthodontics, Faculty of Dental Medicine, Victor Babeş University of Medicine and Pharmacy, 9 RevolutieiBv., Sq., Timişoara 300041, Romania; ^10^Department of Surgical Semiology, Faculty of Medicine, Victor Babeş University of Medicine and Pharmacy, 9 RevolutieiBv., Sq., Timişoara 300041, Romania

## Abstract

Breast cancer is the most frequently diagnosed malignant pathology, representing the primary cause of cancer death in women. Natural products are an appealing strategy to limit the progression of the disease. Targeting angiogenesis in breast cancer may positively impact on poor prognosis of breast cancer. As source of natural compounds, we investigated the leaves of *Melissa officinalis* L. (MO), known as lemon balm, an aromatic plant that spontaneously grows in the South and Western areas of Romania, being traditionally recommended as anxiolytic, antispasmodic, or as digestive remedy. Our aim was to investigate the phytochemical profiling and the antiangiogenic and chemopreventive bioactivity of MO from Banat region, on breast cancer. Two ethanolic extracts of MO (MOE96 and MOE70) and one methanolic extract (MOM80) were subjected to polyphenol and triterpene profiling by HPLC-MS, and the antioxidant capacity was evaluated. The antiangiogenic potential was investigated using the chorioallantoic membrane assay (CAM). The MTT(3-(4,5-dimethylthiazol-2-yl)-2-5-diphenyltetrazolium bromide) assay was used to investigate the cytotoxic effects on MCF-7 and MDA-MB-231breast cancer cells, as well as on MCF-10A normal breast epithelial cells, while apoptosis was performed by DAPI staining. Rosmarinic acid (RA) and ursolic acid (UA) were revealed as dominant phytocompounds. The highest concentration in phytochemicals were found in MOM80; MOE96 was more concentrated in UA, while MOE70 extracted more RA. MOE96 inhibited cancer progression and angiogenesis in the in ovo CAM model using MDA-MB-231 cells, inhibiting breast cancer progression and angiogenesis for the MDA-MB-231 breast cancer cell line; no secondary tumoral areas were registered, indicative for a preventive effect against breast tumor cell invasiveness. The highest cell inhibitory activity was also exhibited by MOE96, in particular against the estrogen receptor positive MCF7 breast cancer cell line, with no cytotoxic effect on healthy cells. The estrogen receptor positive MCF7 cell line proved to be more sensitive to the extract antiproliferative activity than the triple negative MDA-MB-231 breast cancer cell line. Nevertheless, the chemopreventive potential of MOE96 extract is phenotype-dependent and is rather related to the apoptosis and antiangiogenic effects suggesting a multitargeted mechanism of action due to its multiple compound composition next to a concentration ratio of RA : UA in favor of UA.

## 1. Introduction

Breast cancer is an important worldwide public health problem, being the most frequently diagnosed cancer and the first cause of cancer death in women [1]. In recent years, cutting-edge research has contributed to important developments in the diagnosis and treatment of breast cancer, thus reducing its consecutive mortality [2]. Despite significant molecular advances currently exploited in various therapeutic strategies, results are confronted with drug resistance and lack of response which still provide a poor prognosis [3]. Efforts are now focused on finding new drugs with higher selectivity and lower side effects that are able to stop breast cancer progression. Studies indicate that the use of natural products and the adoption of healthy diets are associated with the ability to prevent and even treat breast cancer by targeting different molecular pathways [4, 5]. Medicinal plants are being considered an important source for the discovery of new antitumor agents and many phytocompounds have already been described as promising chemopreventive agents [6]. Widely sought folk medicines used traditionally for certain well-known biological effects represent a good source for new drugs; still, validation of their bioactivities and evidence-based studies are required to confirm their safety and efficacy.

The source of natural compounds investigated here is *Melissa officinalis* L. (MO), a perennial aromatic plant belonging to the *Lamiaceae* family, also called lemon balm due to its lemon scent. The chemical composition consists in low amounts of essential oils, triterpenes, and important concentrations of polyphenols (caffeic acid derivatives as rosmarinic acid and flavonoids) [7, 8]. Although distributed worldwide, the plant commonly grows in the Mediterranean region and Western Asia, being intensively cultivated in Europe [9, 10]. For more than 2000 years, MO has been used as culinary herb and traditionally recommended as sedative, anxiolytic, antistress, antispasmodic, digestive, antimicrobial, and antiviral remedy [11]. The plant was associated with relieving a series of complaints mentioned in early medical references such as Dioscorides (49–90 CE) in De Materia Medica or Paracelsus (1493–1541). Traditional uses from Europe are mainly linked to treating nervous ailments, insomnia, melancholia, and digestive and cardiac symptoms. There are a few records from Islamic Traditional Medicine systems regarding the early use of lemon balm in tumor conditions alone or in combination with other traditional medicines [9]. A more recent ethnopharmacologic study from Turkey revealed the use of aerial parts of *Melissa officinalis* L. in cancer [12]. Moreover, an assessment of the potential risks associated with the use of traditional herbal medicines during conventional chemotherapy in Middle Eastern patients revealed that lemon balm might reduce the activity of tamoxifen, a chemotherapeutic agent commonly used in ER positive breast cancer [13]. MO spontaneously grows in the South and Western areas of Romania which are characterized by a continental climate with sub-Mediterranean influences, where leaves and sometimes flowering tops of the plant are used for the treatment of digestive problems, asthma, and rheumatism [14, 15].

The potential anticancer effects of lemon balm were previously studied using several types of extracts on various tumor cells. Breast cancer, next to lung, prostate, or colon cancer, is among the most investigated types of cancer, particularly *in vitro* [16, 17], but also *in vivo *[18]. Some studies reported that alcoholic extracts were more effective than hydroalcoholic extracts against certain types of tumor cells, such as the hormone dependent MCF7 breast cancer cells [16], while others highlighted the sensitivity of MCF7 cell line to MO hydroalcoholic extracts [7]; in addition, MO aqueous extracts were proven effective against both hormone-dependent (MCF7 cell line) and non-dependent (MDA-MB-468 and MDA-MB-231 cell lines) breast cancers [18]. More recently, molecular mechanisms were investigated [19], all suggesting the high potential of lemon balm as a valuable source of extracts rich in phytocompounds with benefits against cancer progression. Antiangiogenic effects of some lemon balm extracts were also reported, but not in the particular case of breast cancer [20]. Therefore, breast tumor targeting and the active effectors from the leaves of lemon balm are yet to be elucidated. Although from the economical point of view it is more attractive to select and use the purified most active compounds from a plant, this approach involves the elimination of the synergistic biological effect produced by the mixture of compounds found in a plant extract; the use of total extracts provides a gentler and safer biological activity due to the synergistic effects of smaller amounts of active compounds as compared to the strong, brutal activity of a single compound considered as “silver bullet” [21].

Several other natural chemopreventive agents have already been described as therapeutic solution for breast cancer: curcumin, sauchinone, lycopene, denbinobin, genipin, capsaicin, and ursolic acid; these agents act through various mechanisms and may affect both estrogen receptor ER+ and ER− types of breast cancer [6]. Chemoprevention through natural products is an interesting approach in limiting the progression of the breast cancer, by slowing, suppressing, or reversing the carcinogenic process. One of the main hallmarks of poor progression in breast cancer is represented by the deregulated and overactivated angiogenic process [6]. The formation of new vessels is a finely tuned process of the tumor microenvironment, which is excessively stimulated in order to facilitate tumor growth and metastasis [22].

Aiming to repurpose the traditional use of lemon balm, the current research was undertaken in order to investigate the potential angioprevention and chemoprevention in breast cancer of various MO extracts next to the main phytocompounds, as determined by HPLC measurements (rosmarinic acid and ursolic acid). Rosmarinic acid found in high amounts in the plant material [11] is considered an important component of the MO extract, partially responsible for its biological effects [23]. Another phytocompound associated with anticancer effects [24] is ursolic acid, a triterpene also present in the composition of lemon balm [11]. The potential effects on angiogenesis, tumor formation, and development were evaluated for the first time *in ovo*, for breast cancer, using the chorioallantoic membrane assay. The antioxidant activity and *in vitro* effect on cell viability and on the proapoptotic potential were assessed on two breast cancer cell lines, MCF7 (ER+) and MDA-MB-231 (ER−).

## 2. Materials and Methods

### 2.1. Plant Material and Extraction

Fresh leaves of *Melissa officinalis* L. were harvested from Dumbravita, Timis County, in the western area of Romania. Plant material was identified and the voucher specimens were deposited at the Herbarium of the Babes-Bolyai University, Cluj Napoca, Romania (668629). Lemon balm leaves were air-dried at room temperature and grinded prior to be subjected to various extraction techniques using three types of solvents. The powdered dried plant material was weighed and aliquots of 4 grams were treated with 100 ml solvent as follows: one set of samples was obtained by maceration for 9 days in 70% ethanol (MOE70), the second set was obtained by maceration in 96% ethanol for 24 hours under continuous stirring (MOE96), and the third set was extracted by sonication in 80% methanol for one hour (MOM80). Extraction procedures were all performed at room temperature. All samples were filtered and the supernatant was collected in glass vials and stored at 3°C until LC-MS analysis. For the purpose of *in vitro* and *in ovo* analysis, the extracts were concentrated in a rotary vacuum evaporator (Heidolph Laborota 4000, Schwalbach, Germany), and finally freeze-dried extracts were obtained and stored at −20°C prior to use.

### 2.2. Phytochemical Analysis by HPLC-MS

#### 2.2.1. LC-MS Apparatus

High-performance liquid chromatography (HPLC/LC) coupled with mass spectrometry (MS) experiments were conducted on a 6120 LC-MS analytical system from Agilent (Santa Clara, CA, USA) consisting of 1260 Infinity HPLC equipped with G1322A degasser, G1311B quaternary pump, G1316A column thermostat, G1365C MWD detector, and G1328C manual injector coupled with a Quadrupolar (*Q*) mass spectrometer equipped with electrospray ionization source (ESI). LC-MS is connected to a PC running the OpenLAB CDS ChemStation Workstation software to control the instrument and acquire and process LC-MS data.

#### 2.2.2. Chemicals

Methanol (99.9% purity), ethanol (99.9% purity), ammonium acetate (99.9% purity), and acetic acid (99.9% purity) were purchased from Merck (Darmstadt, Germany) and used without further purification. Standard polyphenolic compounds: rosmarinic acid, caftaric acid, gentisic acid, chlorogenic acid, caffeic acid, p-coumaric acid, ferulic acid and sinapic acid, hyperoside, isoquercitrin, rutin, myricetin, fisetin, quercitrin, quercetol, luteolin, kaempferol, and apigenin, were purchased from Sigma-Aldrich (Germany). Standard pentacyclic triterpenic compounds: ursolic, oleanolic, and betulinic acid, were also purchased from Sigma-Aldrich (Germany). For the preparation of sample solutions, ultrapure deionized water was provided by a MiliQ system Milli-Q® Integral Water Purification System (Merck Milipore, Darmstadt, Germany).

#### 2.2.3. LC-MS Methods

Two LC-MS methods were developed and conducted for the screening and quantification of polyphenolic acids and pentacyclic triterpenic compounds expressed in MO extracts described above. Polyphenols were separated on a reverse phase Zorbax Eclipse Plus C18 column (3.0 × 100 mm × 3.5 *μ*) while pentacyclic triterpenic compounds were separated on a reverse phase Ultrasphere ODS C18 column (4.6 × 250 mm × 5 *μ*). The first analytical method enabled the screening and quantification of all extracts for 18 polyphenols (rosmarinic acid, caftaric acid, gentisic acid, chlorogenic acid, caffeic acid, p-coumaric acid, ferulic acid and sinapic acid, hyperoside, isoquercitrin, rutin, myricetin, fisetin, quercitrin, quercetol, luteolin, kaempferol, and apigenin) by an analytical method previously developed [25, 26]. To separate the components, a mixture of 0.1% acetic acid and methanol was used as mobile phase in gradient elution as follows: up 5 minutes 5% methanol, up to 38 minutes 42% methanol and maintained up to 41 minutes, up to 42 minutes 5% methanol. The flow rate was 1 ml/min, the elution of all components was achieved in about 40 minutes, the injection volume was 10 *μ*l, and the column temperature was set at 40°C. The screening of compounds was conducted by both the UV and MS detectors. UV detection was conducted at 330 and 370 nm. MS detection was achieved by electrospray ionization (ESI) in the single ion monitoring mode (SIM) simultaneous for all 18 screened compounds. All mass spectra were recorded in the negative ion mode, previously demonstrated to be the most appropriate option for this type of compounds [27, 28]. During experiments, the capillary voltage was set at 3500 V, the dry gas flow was 12 l/min at 350°C, the nebulizer pressure was kept at 55 psig, and the fragmentor was set at 70. Calibration curves with good linearity were conducted for the quantification of polyphenols in the samples by the external standard method in the 0.05–2 *μ*g/ml range for a six-point plot for each compound. The second analytical method was newly developed by our group for the purpose of the current study in order to screen and quantify the three pentacyclic triterpenic acids: ursolic, oleanolic, and betulinic acid. The mobile phase is a mixture of 1 mM ammonium acetate and methanol (1 : 3 *v*/*v*) in isocratic elution. The flow rate was 1 ml/min, the injection volume was 20 *μ*l, and the column temperature was set at 20°C. UV detection was conducted at 200 and 210 nm. These chromatographic parameters enabled the elution of the three compounds in about 65 minutes, due to their difficult separation, all 3 phytocompunds being isomers. MS detection was achieved by ESI in the SIM mode simultaneous for all screened compounds, and mass spectra were recorded in the negative ion mode. The capillary voltage was set at 3500 V, the dry gas flow was 12 l/min at 350°C, the nebulizer pressure was kept at 60 psig, and the fragmentor was set at 70. Calibration curves for the quantification of pentacyclic triterpenic compounds in the samples were conducted in the 0.05–2 *μ*g/ml range for a seven-point plot for each compound. The *m/z* scale of the mass spectrum was calibrated by use of an external calibration standard ESI Tuning Mix from Agilent (Santa Clara, CA, USA).

#### 2.2.4. Sample Preparation for LC-MS Analysis

All sample solutions were diluted with ultrapure deionized water, were homogenized with a WisdVM-10 vortex mixer (witeg Labortechnik, Germany), and centrifuged for 2 minutes at 10000 rpm in a Thermo Micro CL17 microcentrifuge (Thermo Fisher Scientific, MA, USA). The supernatant was collected and submitted to LC-MS analysis.

### 2.3. Chorioallantoic Membrane Assay (CAM)

The CAM assay is considered an *in vivo* technique that allows the investigation of effects induced on both angiogenesis and tumor process. In brief, the method makes use of fertilized hen (*Gallus gallus domesticus*) eggs and involves egg cleansing with 70% ethanol before incubation at controlled temperature (37°C) and 50% humidity. On the third embryonic day of development (EDD 3), 3-4 ml of albumen was removed, and an opening was cut on the upper side of the eggs on EDD 4 [29]. Macroscopic evaluation was daily performed *in ovo* by means of stereomicroscopy (ZEISS SteREO Discovery.V8, Göttingen, Germany), and all images were registered and processed by Axiocam 105 color, AxioVision SE64. Rel. 4.9.1 Software, (ZEISS Göttingen, Germany), ImageJ (ImageJ Version 1.50e, https://imagej.nih.gov/ij/index.html), and GIMP software (GIMP v 2.8, https://www.gimp.org/). Photographs were analyzed using Angioquant software, an automated image analysis tool [30], which quantifies the effects on the CAM vascularization by measuring the number of complexes, their length, size, and vessel junctions, compared to the control.

#### 2.3.1. Normal Angiogenesis Evaluation

MO extracts were tested in the median concentration of those evaluated *in vitro*. The normal developing CAM between EDD 7–10 was used both to predict the tolerability of MO extracts on normal tissues and to detect possible effects on highly angiogenic blood vessels. From day 0, 0 h (EDD 7) samples were daily applied and the specimens were evaluated. Six groups of samples were tested: (1) control, represented by DMSO 0.5% as solvent control (*v*/*v* in double distilled water); (2) rosmarinic acid 50 *μ*M (RA 50 *μ*M), as polyphenol standard compound; (3) ursolic acid 50 *μ*M (RA), as triterpene standard compound; (4) MO methanolic extract 50 *μ*g/mL (MOM80); (5) MO 70% ethanolic extract 50 *μ*g/mL (MOE70); and (6) MO 96% ethanolic extract 50 *μ*g/mL (MOE96). Volumes of 5 *μ*l/egg of all samples were applied directly inside plastic rings (3 mm in diameter) previously placed on top of the CAM.

#### 2.3.2. Tumor Angiogenesis Evaluation

In order to assess the effects of MO extracts *in ovo* on the development of breast cancer, a tumor CAM assay was used by the inoculation of breast cancer cells on top of the developing membrane on EDD 10 (day 0, 0 h). The two breast cancer cell lines, MCF-7 and MDA-MB-231, were cultured according to the above described protocol and subsequently inoculated onto the CAMs in a similar manner to our previously published research [31]. Cells detached from the culture plate by trypsinization were cleansed and resuspended in the culture medium until reaching the final concentration of 10^5^ cells/5 *μ*L. On EDD 10, 5 *μ*L of breast cancer cell suspension was inoculated inside a plastic ring previously placed onto the membrane. At that point, 5 *μ*L of each sample was applied, as previously described for the evaluation of the normal angiogenic process, divided in six groups, with the control group being represented by cancer cells treated with DMSO 0.5%. Significant images were captured daily *in ovo* and *ex ovo* on the final day of the experiment.

### 2.4. Antioxidant Activity Using the DPPH Assay

The antioxidant activity of *Melissa officinalis* extracts was evaluated by measuring their ability of scavenging free stable DPPH (2,2′-diphenyl-1-picrylhydrazyl) radicals. The determination is based on the DPPH reduction by the hydrogen donating antioxidants, which leads to the discoloration and, subsequently, to the decrease of solution absorbance. The method was carried out as previously described [32, 33], with slight modifications. In brief, 1.8 mL of freshly prepared 0.1 mM DPPH in ethanol was added to 200 *μ*L of each sample in concentration of 50 *μ*g/mL for the lemon balm extracts and 50 *μ*M for the pure compounds and ascorbic acid (AA) as control. The mixture was incubated in the dark for 30 minutes, at room temperature. Absorbance was spectrophotometrically measured at 517 nm against blank samples. The decrease in the registered absorbance indicates a free radical scavenging activity. The antioxidant activity (AOA) is calculated as the scavenging capacity of free DPPH radical (in percentages) using the following formula: AOA (%) = (*A*_0_ – *As*)/A_0_ × 100, where *A*_0_ = absorbance of the blank sample and *As* = absorbance of the tested samples.

### 2.5. Cell Proliferation by MTT Assay

#### 2.5.1. Cell Culture

The human breast adenocarcinoma cell lines MCF-7 (ATCC® HTB-22) and MDA-MB-231 (ATCC® HTB-26) and the breast epithelial cells MCF-10A (ATCC® CRL-10317) were acquired from the American Type Culture Collection (ATCC). MCF7 cells were cultured in Eagle's Minimum Essential Medium (EMEM; ATCC) and MDA-MB-231 cells were cultured in high glucose Dulbecco's Modified Eagle's Medium (DMEM; Sigma-Aldrich), whereas MCF-10A cells were cultured in 1 : 1 mixture DMEM: F-12 medium (ATCC) supplemented with 20 ng/mL epidermal growth factor (EGF; Gibco, Thermo Fisher Scientific), 0.01 ng/mL insulin (Sigma-Aldrich), 500 ng/mL hydrocortisone (Sigma-Aldrich), and 5% fetal bovine serum (FBS; Gibco, Thermo Fisher Scientific). Tumorigenic cell lines were supplemented with 10% FCS. 1% penicillin/streptomycin mixture (Pen/Strep, 10,000 IU/ml; Sigma-Aldrich) was added in each cell culture medium to avoid a possible fungal/microbial contamination. Standard conditions were used for cell culture—37°C and humidified atmosphere containing 5% CO_2_, as previously described before [34, 35].

#### 2.5.2. MTT Assay

The colorimetric microculture tetrazolium assay (MTT) was used to study the viability of MCF-7, MDA-MB-231, and MCF-10A cells in accordance with Mosmann [36]. The cells were seeded in 96-well culture plates at a cellular density of 1 × 10^4^ cells/well and allowed to attach to the bottom of the well. The cells were treated with various concentrations of the tested samples—25, 50, and 100 *μ*M, respectively, for the standard compounds (rosmarinic acid—RA and ursolic acid—UA) and 25, 50, and 100 *μ*g/ml, respectively, of the *Melissa officinalis* L. extracts (dissolved in dimethyl sulfoxide—DMSO; Sigma-Aldrich) and incubated for 24 h. The control group is represented by cells treated with DMSO—the solvent used for sample preparation. Cells were then assayed by the addition of 10 *μ*L of 5 mg/mL 3-(4,5-dimethylthiazol-2-yl)-2,5-diphenyltetrazolium bromide (MTT) solution from the MTT-based *in vitro* toxicology assay kit (Sigma-Aldrich). Intact mitochondrial reductase converted and precipitated MTT as blue crystals during a 3 h contact period. The precipitated crystals were dissolved in 100 *μ*L of lysis solution provided by the manufacturer. Finally, the reduced MTT was spectrophotometrically analyzed at 570 nm, using a microplate reader (xMark Microplate Spectrophotometer, Bio-Rad). The percentage of cell viability was calculated using the formula: cell viability (%) = 100 − [(*A*_0_ − *A*_s_)/*A*_0_ × 100], where *A*_0_ = absorbance of blank sample and *A*_s_ = absorbance of tested samples.

### 2.6. DAPI (4′,6-Diamidino-2-Phenylindole) Staining

Detection of the state of MCF-7 and MDA-MB-231 cells' nuclei condensation was assessed by the means of DAPI, a nuclear stain able to emit blue fluorescence of the live cell nuclei and bright blue fluorescence of apoptotic cell nuclei, upon UV excitation. The cells were seeded on coverslips in 6-well plates until the optimal confluence was reached, followed by 24 h treatment with MO extracts (50 and 100 *μ*g/mL), RA and UA standard compounds (50 and 100 *μ*M), and DMSO used as negative control. DAPI staining was performed as previously described [37], respecting the essential steps: cells were fixed (4% paraformaldehyde in PBS), permeabilized (2% Triton X-100) (Sigma-Aldrich), blocked (30% FCS in 0.01% Triton X-100), and stained with DAPI (4′,6′-diamidino-2-phenylindole) (Sigma-Aldrich) for 15 min in a dark chamber. Cell nuclei were examined at magnification of 40x using an Olympus IX73 fluorescence microscope documented with an integrated DP74 camera (Olympus, Tokyo, Japan).

### 2.7. Statistical Analysis

All experiments were performed in triplicate. Results are presented as mean ± standard deviation (SD). One-way ANOVA analysis followed by Tukey's post hoc test was performed to detect statistical differences among the tested groups *in the case of MTT viability results, while Dunnett*'*s post hoc test was used for the angiogenesis quantification*. *p* values of ^*∗*^*p* < 0.05, ^*∗∗*^*p* < 0.01, ^*∗∗∗*^*p* < 0.001, and ^*∗∗∗∗*^*p* < 0.0001 were considered as statistically significant. Statistical analysis was performed using GraphPad Prism 7 (GraphPad Software, La Jolla, CA, USA).

## 3. Results and Discussion

### 3.1. Phytochemical Analysis by LC-MS

**Figure 1:**
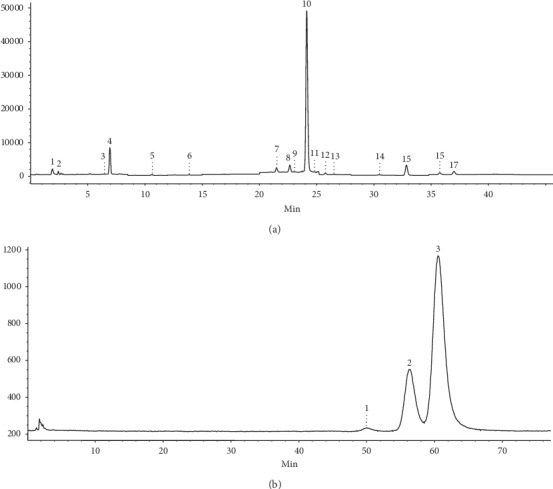
(a) SIM LC-MS polyphenolic profile of MOE70 sample: 1, caftaric acid; 2, gentisic acid; 3, chlorogenic acid; 4, caffeic acid; 5, p-coumaric acid; 6, ferulic acid; 7, hyperoside; 8, isoquercitrin; 9, rutin; 10, rosmarinic acid; 11, myricetin; 12, fisetin; 13, quercitrin; 14, quercetol; 15, luteolin; 16, kaempferol; 17, apigenin. (b) SIM LC-MS of pentacyclic triterpenic compounds screened in MOE96 sample: 1, betulinic acid; 2, oleanolic acid; 3- ursolic acid.

All samples were subjected to LC-MS analysis which enabled the identification and quantification under identical solution and instrumental conditions of two major classes of secondary metabolites in MO: polyphenols and pentacyclic triterpenic compounds [9]. All identified and quantified polyphenolic phytocompounds in the three types of extracts are listed in Table 1 and expressed as *μ*g of phytocompound per gram dry weight (d.w.). Also, the SIM MS spectra in the negative ion mode of the polyphenolic profile of MOE70 sample are depicted in Figure 1(a).

**Figure 2:**
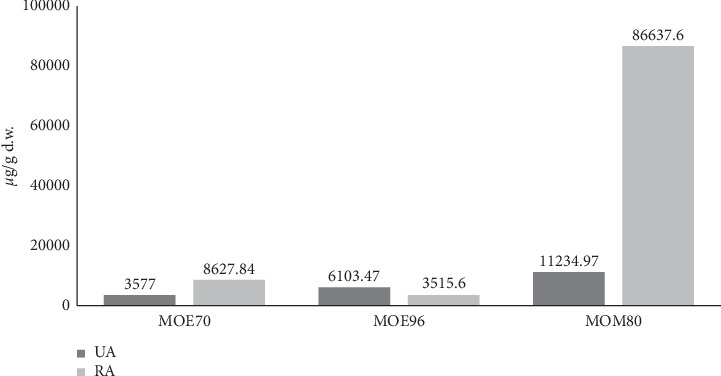
Comparative assessment of RA and UA content in MO extracts: MOE70, MOE96, and MOM80 (*μ*g/g d.w.).

Inspection of the obtained results reveals that, irrespective of the type of extraction, rosmarinic acid (RA) was found to be the most abundant polyphenolic compound. These findings are consistent with many literature reports [8, 10, 38], while others reported different polyphenolic compounds such as rutin to be most abundant [39], or in some cases, MO extracts were not screened for RA at all [40]. In all three extracts, our analytical method enabled the identification and quantification of various amounts of polyphenolic phytocompounds such as caffeic acid, caftaric acid, rutin, and isoquercitrin, as well as small amounts of other compounds (e.g., fisetin, quercetol, and p-coumaric acid). The comparative assessment indicated major differences in terms of RA concentration in the three types of extracts. Thus, methanol sonication yielded concentrations of RA 10 times higher than 70% ethanol maceration (86637 vs. 8627 *μ*g/g d.w.) and almost 25 times higher than 96% ethanol maceration (86637 vs. 3515 *μ*g/g d.w.) (Figure 2).

The use of methanol generally favored the extraction of all polyphenolic compounds to an average increase in concentration of 500% when compared to ethanolic extraction, but in some cases (e.g., isoquercitrin), the difference can reach double the average. Methanol combined with 30 minutes sonication was previously found to be the most favorable method for polyphenolic compounds extraction [41], but the procedure generated various results presumably due to the particular environmental factors that influence the MO phytochemical profile. Thus, it seems that MO from western Romania is much richer in RA (2 to 10 times higher) than plants originating from Poland, but at the same time poorer in other polyphenols such as ferulic acid [41]. Interesting differences in polyphenolic acid expression can also be spotted out when comparing the two ethanolic solvents. With one exception, represented by rutin, MOE70 yielded concentrations several times higher in phytocompounds than MOE96, presumably due to the chemical peculiarities of polyphenols and also the longer period of time in which the plant material was in contact with the solvent (9 vs.1 day). Rutin seems to exhibit a higher solubility and extraction yield in 96% ethanolic solution rather than in 70% ethanol. One can also notice that, depending on the extraction technique, some compounds were only detected but not quantified, falling below the quantification limit (e.g., kaempferol, luteolin, or chlorogenic acid); sinapic acid was not identified in any of the extract sample.

Next, the phytochemical characterization of MO extracts continued with the screening and quantification of all samples for pentacyclic triterpenic compounds; detected and quantified compounds in MOE70, MOE96, and MOM80 are listed in Table 2. The SIM MS spectra recorded in the negative ion mode of MOE70 sample is depicted in Figure 1(b).

The examination of obtained data discloses that all MO samples contain UA, OA, and BA in different amounts. Limited data are available regarding the content of triterpenes in MO; nevertheless, our findings are consistent with previous reported results that mainly identified UA and OA [42–44], while only one research group detected the presence of BA in MO extracts [45]. Out of the three pentacyclic triterpenic compounds, UA was the dominant component in terms of concentration, as reported before [42], regardless of the type of extract, followed by OA and smaller amounts of BA. The comparative assessment of MOE70, MOE96, and MOM80 extracts reveals that methanol sonication favored UA extraction, thus yielding concentrations more than 3 times higher than the 70% ethanol maceration (11234 vs. 3577 *μ*g/g d.w.) and almost double than the 96% ethanol maceration (11234 vs. 6103 *μ*g/g d.w.) (Figure 2). Similar to the first class of analyzed compounds, MOM80 is the richest extract in all three pentacyclic triterpenic phytocompounds, clearly indicating that 80% methanol is the most effective type of extraction solvent for this class of compounds. When compared to ethanolic extraction, one can notice that BA exhibits the greatest difference in terms of solubility in the selected solvents, most probably related to its poor water solubility; thus, MOE80 is 10 times richer in BA than MOE96. OA is also more soluble in methanolic solution, displaying concentrations more than 4 times higher in MOM80 than in MOE96. In spite of a shorter maceration period, MOE96 sample is 30% more concentrated in BA, 60% in OA, and over 70% in UA than MOE70. Our newly developed LC-MS analytical method enabled for the first time the simultaneous screening and quantitation of UA, OA, and BA from MO extracts, while our different extraction strategies combined with LC-MS analysis revealed the most suitable solvent/method to reveal the presence of these types of phytocompounds in MO.

### 3.2. Antiangiogenic Effects *In Ovo* Using the CAM Assay

**Figure 3:**
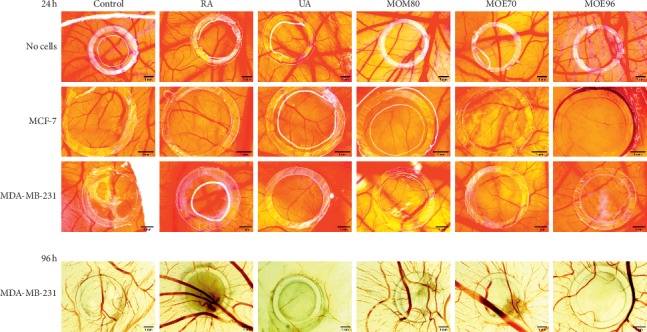
Effects on the CAM assay for MO extracts (50 *μ*g/ml) and control standard compounds RA and UA (50 *μ*M): 24 h poststimulation on normal developing CAM (no cells) following the inoculation of MCF-7 and MDA-MB-231 breast cancer cells; 96 h poststimulation samples were evaluated *ex ovo* for the MDA-MB-231 cells on CAM. The scale bars represent 1 mm.

As far as we know, this is the first evaluation of lemon balm ethanolic or methanolic extracts on the CAM assay, regarding their potential effects on the angiogenesis process involved in breast cancer progression. The suppression of the angiogenesis process is known as an antitumor and antimetastatic mechanism [31]. We investigated the potential antiangiogenic effect of the three MO extracts (50 *μ*g/ml), next to the two pure compounds, RA and UA (50 *μ*M). Firstly, we assessed the general tolerability and the effect on the normal developing chorioallantoic membrane, 24 hours following treatment (Figure 3). From our previous evaluation, we recorded that the 50 *μ*g/mL concentration of extracts was well tolerated, with high survival rates on medium and long-term assessment (data not shown). When comparing the six groups of samples—one control and five treatment groups—experimental data showed that the MOE70 extract was best tolerated with no modification induced on the normal angiogenic process. When compared to MOM80, MOE96 showed a slightly higher vessel density, though none of the samples induced toxicity on the vascular architecture and functionality. In case of the two standard compounds used in 50 *μ*M concentration, ursolic acid induced a slight reduction of the vascular density, while rosmarinic acid did not influence the normal CAM vascular plexus. Collectively, these data indicated the lack of significant toxicity of pure compounds and total extracts as well, when applied on normal tissues.

Secondly, we assessed the effects induced by the tested samples onto the developing CAM after the inoculation of MCF-7 and MDA-MB-231 breast cancer cells. The follow-up was performed 24 hours posttreatment of CAMs already inoculated with tumor cells and 96 hours posttreatment as well (Figure 3). Secondary areas of tumor cells outside the application ring were not observed for any of the tested groups, signaling that both pure compounds or multicompound extracts exert a preventive effect against breast tumor cell invasiveness.

**Figure 4:**
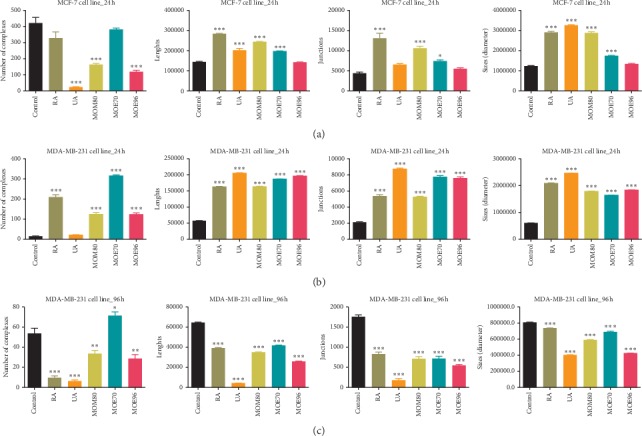
Angiogenesis quantification using the CAM assay for MO extracts (50 *μ*g/ml) and control standard compounds RA and UA (50 *μ*M): 24 h poststimulation of MCF-7 (a) and MDA-MB-231 breast cancer cells (b); 96 h poststimulation for the MDA-MB-231 cells on CAM (c); number of tubular complexes, total length, size, and number of junctions of the blood vessels were measured using the Angioquant software on the recorded photographs. Experiments were performed in triplicate and data are presented as mean ± SD, *n* = 3; ^*∗*^*p* < 0.05; ^*∗∗*^*p* < 0.01; ^*∗∗∗*^*p* < 0.001 vs. control, calculated by the one-way ANOVA test followed by Dunnett's post hoc test.

When comparing the modifications induced after 24 hours of incubation, the antiangiogenic effect is observed only on the MCF-7 cells in terms of reduced number of complexes and by all samples except MOE70. Analyzing the effect after 24 hours of incubation on the MDA-MB-231 cells, an initial amplification of the angiogenic parameters was registered except for a low effect of UA on the reduction of the number of vascular complexes (Figures 3 and 4). Although 24 hours posttreatment, the antiangiogenic effect was reduced, when comparing the test groups, we noted that RA and two of the extracts with the higher ratio of RA : UA (*i*.*e*., MOM80 and MOE70) induced more important reduction in all angiogenic parameters, except the number of complexes, for the MDA-MB-231 cell line (Figure 4). The experiment for the ER− breast cell line follow-up was also done after 96 h posttreatment. At this point, the most potent antiangiogenic effect was registered for the MOE96 extract on the MDA-MB-231 cell line, in correlation with the strongest effect of UA that exceeds RA in this particular type of extract (RA : UA, 1 : 2).

The overall observation was that MOE96 induced the strongest effect in terms of reducing tumor cell growth, in direct correlation with the *in vitro* results. This type of extract induced rapid decrease of cell proliferation, showing a low number of scattered cells inside the application ring and a lower number of new blood vessels. The antiangiogenic effect induced by MOE96 24 hours posttreatment was only detected on MCF7 cells, by reducing the number of tubular complexes; however, 96 hours posttreatment, a more pronounced antiangiogenic effect was recorded for the MDA-MB-231 cells inoculated on CAMs, in terms of number of tubular complexes, length, diameter, and also branching pattern (Figures 3 and 4).

Interesting differences were reported regarding MOM80 and MOE70 extracts in relation with the two types of cell lines. MOM80 exerted a stronger inhibitory effect on tumor development and on the number of tubular complexes in MCF-7 inoculated specimens, an effect that appeared 24 hours posttreatment. The methanolic extract MOM80 induced a scattered aspect of the MCF-7 cells, while the same extract induced a compact display of tumor cells with a spoke-wheel angiogenic pattern in the peritumoral area of MDA-MB-231 after 96 hours (Figure 3), which was also expressed in a higher value of the number of junctions (Figure 4) quantified by the Angioquant software. The less active *in vivo* extract in terms of cell growth limitation and compact tumor formation was MOE70; it was less tolerated on the MCF-7 inoculated specimens and induced a relatively more extended tumor cell area, but without an important impact on angiogenesis. When tested on the MDA-MB-231 cells inoculated on CAM, MOE70 moderately influenced cell adherence, with the formation of some peritumorally vascularized compact areas, with values expressing the number of complexes surpassing the control specimen and all the other samples as well (Figure 4).

The pure tested compounds had different effects on the two types of breast cancer cells and, generally, were less active against tumor formation, acting as antiangiogenic compounds especially 96 hours postinoculation with MDA-MB-231 cells. The exhibited effects of pure RA and UA, respectively, were different on tumor cells and on the angiogenic process. RA induced a scattered aspect of the estrogen receptor positive MCF-7 cells, while more compact groups of cells was noticed in the case of triple negative MDA-MB-231 cells, indicative for a possible impairment of the MCF-7 cell adhesion ability; RA did not inhibit the tumor angiogenic process of MCF-7 cells after 24 hours, but reduced all angiogenic measured parameters 96 hours posttreatment for the MDA-MB-231 cells. UA had a more pronounced effect on tumor angiogenesis, reducing the number of blood vessel complexes (Figure 4) for both cancer types. The total extracts, in particular MOE96, exhibited more important reduction of the angiogenesis process compared to RA on both cell lines after short and long-time stimulation, while reducing the size of the blood vessels comparable to the effect of 50 *μ*M UA, a higher concentration than that present in the tested extracts.

Previous evaluations regarding the possible angiogenic implications of lemon balm extracts were performed only *in vitro* on HUVEC endothelial cell lines and *in vivo* on mouse models. Aqueous extracts were found to decrease VEGF-induced angiogenesis (vascular endothelial growth factor) in a dose related manner [46], while hydroethanolic extracts reduced VEGF-A, increased TSR (thrombospondin type 1 repeat) mRNA expression in mice, and inhibited the capillary-like formation in HUVEC cell line [20]. The antitumor effect of MO ethanolic extracts on prostate, colon, and breast cancer seems to be in part due to the downregulation of VEGF-A and hTERT (telomerase reverse transcriptase) as regulators of carcinogenesis [19]. CAM-based tumor models represent an important future approach in cancer studies, being a useful alternative for preclinical research, benefiting from low cost, little time-consuming, and versatility in studying the early effects or even the resistance against anticancer and antiangiogenic therapeutics [47].

### 3.3. Antioxidant Activity

**Figure 5:**
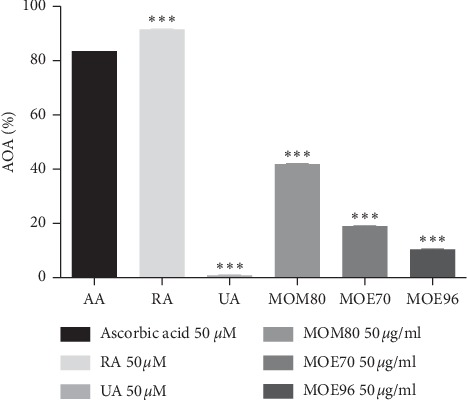
Antioxidant activity of *Melissa officinalis* L. extracts in concentration of 50 *μ*g/mL, (MOM80, MOE70, and MOE96), next to rosmarinic acid (RA) and ursolic acid (UA) and ascorbic acid (AA) as control, in concentration of 50 *μ*M; values (mean ± SD, *n* = 3) were expressed as percentage (%) of scavenged free DPPH radicals; ^*∗∗∗*^*p* < 0.001 vs. AA as control, calculated by one-way ANOVA followed by Tukey's post hoc test.

The antioxidant activity (AOA) was evaluated for the three types of MO extracts (50 *μ*g/mL) next to standard compounds RA (50 *μ*M, corresponding to 18 *μ*g/mL) and UA (50 *μ*M, corresponding to 22.8 *μ*g/mL) and compared to ascorbic acid (AA, 50 *μ*M, corresponding to 8.8 *μ*g/mL) as control (Figure 5). All three extracts showed an antioxidant activity above 10%. The highest value was recorded for the methanolic extract, MOM80 (41.9 ± 0.156%), followed by the 70% ethanolic extract, MOE70 (18.96 ± 0.156%), while the lowest values were registered by the 96% ethanolic extract, MOE96 (10.36% ± 0.156). Controversially, the pure standard compounds showed quite different antioxidant potential. RA surpassed the ascorbic acid antioxidant potential, while the same concentration of UA shows a very limited antioxidant effect. Our results indicated an AOA of 91.51% ± 0.156 for RA, and only 0.76% ± 0.156 for UA, data that are consistent with previously published papers which reported the following IC_50_ for the AOA in DPPH assay: IC_50_ for UA∼5 mg/mL > IC_50_ for AA∼15 *μ*g/mL > IC_50_ for RA = 5.5 *μ*g/mL [48–50]. Hence, the antioxidant potential of the three MO extracts was comparatively evaluated by taking into consideration their respective concentrations in RA, known as a strong antioxidant compound [17]. The lemon balm extract with the highest concentration in RA (MOM80 86.6 mg/g d.w.) expressed the highest antioxidant capacity, followed by MOE70 (8.6 mg/g d.w.) and MOE96 (3.5 mg·RA/g d.w.); thus, a direct correlation between the extract content in RA and its antioxidant capacity could be established.

### 3.4. Cell Viability Effect against MCF-10A Breast Epithelial Cells and MCF-7 and MDA-MB-231 Breast Cancer Cells

**Figure 6:**
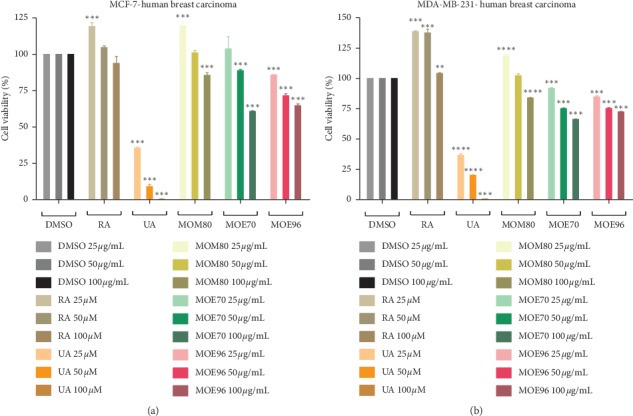
Cell viability effect of MO extracts (25, 50, and 100 *μ*g/mL) and standard compounds RA (25, 50, and 100 *μ*M, corresponding to 9–36 *μ*g/mL) and UA (25, 50, and 100 *μ*M, corresponding to 11.4–45.6 *μ*g/mL), determined by the MTT assay, 24 h poststimulation of MCF-7 (a) and MDA-MB-231 (b) breast cancer cells. Results are represented as percentage (%) of viable cells compared to DMSO-negative control. Results are presented as mean ± SD (*n* = 3); ^*∗*^*p* < 0.05; ^*∗∗*^*p* < 0.01; ^*∗∗∗*^*p* < 0.001, ^*∗∗∗∗*^*p* < 0.0001 vs. control, calculated by one-way ANOVA followed by Tukey's post hoc test.

The three MO extracts and standard compounds, RA and UA, were evaluated in terms of their effect on cell viability on two breast carcinoma cell lines, MCF-7 and MDA-MB-231, the former representing an ER+ type of breast cancer and the latter a triple negative breast cancer. The effect of the tested samples on MCF-7 and MDA-MB-231, respectively, after 24 h stimulation is shown in Figures 6(a) and 6(b). Obtained results indicate that the standard compounds, tested in concentrations of 25–100 *μ*M (corresponding to 9–36 *μ*g/mL·RA and 11.4–45.6 *μ*g/mL·UA), exhibited an opposite effect on the cell viability of the two breast cancer cell lines; on both breast cancer cells, ursolic acid induced a significant decrease of cell viability (below 50% even at the lowest concentration, 25 *μ*M) while RA showed a complete lack of inhibitory activity or even stimulated cell proliferation, in particular on the MDA-MB-231 cell line. The inhibitory potency of ursolic acid was similar on the two tested cancer cell lines.

Although considered as a valuable potential chemotherapeutic agent in terms of arresting cancer progression, the IC_50_ values previously reported for UA on the two breast cancer cell lines are controversial, ranging from approximately 1 *μ*M to 500 *μ*M [24, 51]. The anticancer activity of UA used in low micromolar concentrations (5–20 *μ*M) on two phenotypically distinct breast cancer cell lines (MCF-7 and MDA-MB-231) was attributed to the changes induced by UA to the glycolytic pathway leading to cytotoxic autophagy as well as apoptotic cell death [52]. Another study indicated that UA-induced autophagy and apoptosis reduce the viability of breast cancer cells without causing cell death; also, UA is able to suppress the invasive ability of breast cancer cells and reduce inflammation, thus preventing the progression of breast cancer [24].

With regard to rosmarinic acid tested as pure compound, our results are consistent with other previously published studies showing that at 50 *μ*g/mL, RA increased cell viability of breast cancer cells, in particular estrogen-dependent cells (MCF-7) [53]. However, data regarding the cytotoxic effect of RA remain controversial due to other studies that reported cell inhibitory effects for RA on the MCF-7 cell line and indicated IC_50_ values ranging from as low as 2 *μ*M to 200 *μ*M [23].

When evaluating the three types of lemon balm extracts, tested in concentration of 25–100 *μ*g/mL, on the two breast cancer cell lines, respectively, the first observation to be noted is that all three extracts exhibited an inhibitory activity against the two cancer cell lines in a dose-dependent manner, in particular MOE70 and MOE96; MOM80 showed a weak inhibitory activity, only when used in the highest tested concentration, 100 *μ*g/mL, the lower concentrations exhibiting either a lack of activity (50 *μ*g/mL) or even inducing cell proliferation (25 *μ*g/mL). A slightly stronger inhibitory activity was registered for both MOE70 and MOE96 against the ER+ MCF-7 cell line. Their effect on cell viability can be perfectly correlated with their concentrations in the pure compounds, RA and UA. MOM80 contains the highest concentration in both compounds (86637.6 *μ*g/g d.w. RA and 11234.97 *μ*g/g d.w. UA, see Tables 1 and 2), so one would expect it to act as the most potent inhibitory extract of the three currently analyzed; however, the ratio of RA versus UA concentration is approximately 8 : 1 which indicates that the proliferative activity of RA will prevail to the cytotoxic effect of UA. Therefore, in spite of the high content of active principles, MOM80 cannot produce significant inhibitory effects unless applied in high concentrations (100 *μ*g/mL). The excess of RA as compared to UA is maintained in MOE70 which contains 8627.84 *μ*g/g d.w. RA and 3577 *μ*g/g d.w. UA; however, in this case, the RA : UA concentration ratio becomes 2.4 : 1, indicating that UA may strongly exert its inhibitory effect. Indeed, for MOE70, the lowest concentration did not affect cell viability or even induce a slight stimulatory effect, while higher concentrations significantly decreased cell viability in a dose-dependent manner. As one can see, the median dose, 50 *μ*g/mL, inhibited in particular the estrogen-dependent cell line MCF-7 while the highest dose of MOE70, 100 *μ*g/mL, had a similar effect against both breast cancer cell lines. For MOE96, the RA : UA concentration ratio is reversed, becoming 1 : 2 (3515.6 *μ*g/g d.w. RA and 6103.47 *μ*g/g d.w. UA); this change is reflected in its effect on cell viability. All of the three concentrations show an inhibitory effect on both cell lines, also in a dose-dependent manner, with a slightly higher potency against the MCF-7 cell line; in this case, the inhibitory activity of ursolic acid prevailed to the proliferative activity of RA.

If we compare the cell inhibition induced by pure UA versus MOE96, a superficial look might indicate that the use of UA is clearly preferable due to its much stronger cytotoxic effect; however, the two respective concentrations in UA must be taken into account when comparing the two biological activities. While pure UA was applied in a concentration range of 25–100 *μ*M, its concentration in the used extracts was much lower: for example, 100 *μ*g/mL MOE96 extract contains 0.35 *μ*g/mL·RA and 0.61 *μ*g/mL UA, corresponding to a concentration of 0.97 *μ*M and 1.34 *μ*M, respectively. Therefore, we may state that we achieved 30% cell inhibition for both types of breast cancer when using a very small concentration of 1.34 *μ*M active UA (in the form of MOE96 extract), even in the presence of a significant amount of pro-proliferative RA (0.97 *μ*M), while a much higher UA concentration (25 *μ*M) achieved cell inhibition of only approximately 50%; this phenomenon may be the result of a multitargeted activity that might occur due to UA and other compounds such as oleanolic or betulinic acid also present in the extract in smaller amounts, as indicated by the HPLC analysis. The synergistic activity exhibited by oleanolic and ursolic acids was previously documented [54, 55] by *in vitro* studies on two human melanoma cell lines (A375 and A2058). RA is also capable of inducing synergistic effects in spite of its lack of cytotoxicity; in 2017, Bahri et al. reported a significant decrease of the IC50 value for the combination of RA and carnosic acid compared to carnosic acid alone, while RA alone had no cytotoxic effect on human lung fibroblasts [56]. Their data indicated an antifibrotic effect of the combination of the two compounds due to a synergistic proapoptotic activity.

Another possibility may consist in the sensitization of the two breast cancer cell lines to the cytotoxic activity of UA by the presence of the large plethora of polyphenolic compounds revealed by the HPLC assessment. The use of polyphenols as sensitizers was reported by Singh et al. on MCF-7 and MDA-MB-231 cell lines; the combination of low doses of polyphenols with cytotoxic compounds augmented the apoptotic activity of the active drug while providing the advantage of reduced side effects [57].

Several other research groups studied the potential chemopreventive effect of the abundantly available and frequently used medicinal plant, *Melissa officinalis* L.; the results varied in a large range, as a great variability lays in the extraction procedure, next to the geographical source of plant material. Some studies reported that aqueous Soxhlet extracts of MO from Turkey may affect breast cancer cell viability, with IC_50_ values of 18 *μ*g/mL against MCF-7 and 19 *μ*g/mL against MDA-MB-231 [18]. Our previous study conducted on a single 70% ethanolic extract from the lemon balm leaves showed an IC_50_ of 196 *μ*g/mL for the triple negative cells MDA-MB-231 [58]. A more recent paper regarding lemon balm collected in Portugal reported lower IC_50_ values for the ethanolic extracts (122 *μ*g/mL), compared to the methanolic ones (181 *μ*g/mL), despite their higher content in polyphenolic compounds. The same study indicated that hydroalcoholic extracts had intermediate effects, while the aqueous decoctions were less active [16]. Our results confirm the cytotoxic activity previously reported for lemon balm ethanolic extracts on cancer cells but indicate significant antiproliferative activity at much smaller doses than previously used, in the form of ethanolic extracts. Additionally, we compared the effects on the two different types of breast cancer cells, observing a slightly more significant reduction in cell viability exerted on the estrogen receptor positive MCF-7 cells, as previously reported [7].

The cell inhibitory activity is in particular worth noticing on the ER− cancer cell line MDA-MB-231; while the ER+ cell lines such as MCF-7 are sensitive to treatment with hormone and other targeted therapies, triple negative breast cancers such as MDA-MB-231 do not express any receptors and can be therefore subject only to cytotoxic chemotherapy [59]. MDA-MB-231 cancer cells are considered more aggressive than MCF-7 cells and may acquire resistance to chemotherapeutic agents due to the overexpression of a nuclear factor kappa B- (NF-*κ*B*-*) related gene that leads to a higher aggressiveness and cell resistance [60]; these findings may partially explain the small difference in cell inhibition induced by MO extracts on the two breast cancer cell lines (higher cytotoxic activity against MCF-7 cells).

When confronting the results attained by the pure compounds and MO extracts on cell viability and the antioxidant capacity, an inverse relationship can be noticed. MOE96 (the extract with the highest concentration in UA) and UA exert the strongest effect on the reduction of cell viability, while exhibiting the lowest AOA, thus implying that extract effect may be due to the UA contained.

**Figure 7:**
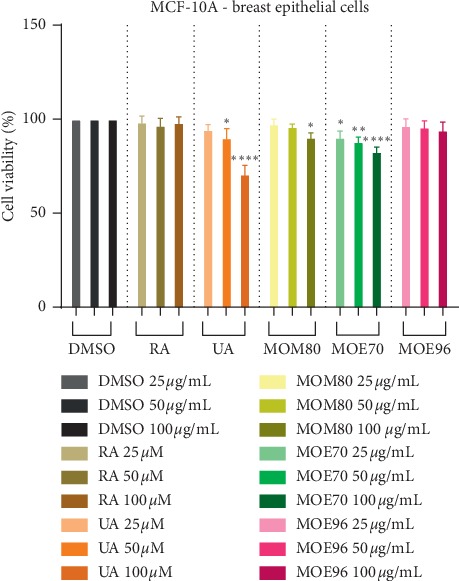
Cell viability effect of MO extracts (25, 50, and 100 *μ*g/mL) and standard compounds RA (25, 50, and 100 *μ*M, corresponding to 9–36 *μ*g/mL) and UA (25, 50, and 100 *μ*M, corresponding to 11.4–45.6 *μ*g/mL), determined by the MTT assay, 24 h poststimulation of MCF-10A cells. Results are represented as percentage (%) of viable cells compared to DMSO-negative control. Results are presented as mean ± SD (*n* = 3); ^*∗*^*p* < 0.05; ^*∗∗*^*p* < 0.01; ^*∗∗∗*^*p* < 0.001, ^*∗∗∗∗*^*p* < 0.0001 vs. control, calculated by one-way ANOVA followed by Tukey's post hoc test.

When assessing the cytotoxic activity of synthetic or natural compounds, an important issue is their selectivity, namely, their ability to exert an antiproliferative effect against cancer cells while leaving healthy cells unharmed. In this regard, we tested the biological activity of pure compounds and extracts also on breast epithelial MCF-10A cells (Figure 7). One can clearly notice that RA did not affect cell viability, not even when used in the highest dose; the effect recorded for UA was not similar. The highest dose, 100 *μ*M, affected MCF-10A cell population, displaying a cell viability percentage of 70.86%. Simultaneously, the best tolerated extract in all applied concentrations was MOE96, followed by MOM80 and MOE70. When corroborating the *in vitro* results obtained on both cancer and healthy cell lines, we may conclude that in spite of the strong antiproliferative activity exerted by UA against the two breast cancer cell lines, the compound cannot be considered as the optimal solution for cancer treatment due to its lack of selectivity. At the same time, MOE96 extract exhibited a significant cytotoxic activity against both breast cancer cell lines while lacking any negative effect on the normal MCF-10A cell line, thus showing a high selectivity degree. MOM80 also lacked cytotoxic effect on the normal cell line but its *in vitro* antitumor activity was also negligible. The second most active antiproliferative extract, MOE70, exhibited a moderate cytotoxic effect against normal cells, in particular in the highest concentration, 100 *μ*M, displaying a cell viability percentage of 82.77%. The samples were also screened in regard to their cytotoxic potential on another normal cell model, using immortalized human keratinocytes—HaCaT cells. Results were similar to the ones obtained on MCF-10A cell, mentioning that the HaCaT cells seemed more sensitive, especially after treatment with the highest dose, 100 *μ*M, of UA (data not shown). This cytotoxic activity of UA against HaCaT cells was previously documented by Harmand et al. who reported a decrease of cell viability in a time and dose-dependent manner through caspase-3-mediated apoptosis and cell cycle arrest [61]. Collectively, these data recommend the use of MOE96 as a potent and selective antitumor agent in breast cancer.

### 3.5. Apoptosis Assessment of Breast Adenocarcinoma MCF-7 and MDA-MB-231 Cells via DAPI (4′,6-Diamidino-2-Phenylindole) Staining

**Figure 8:**
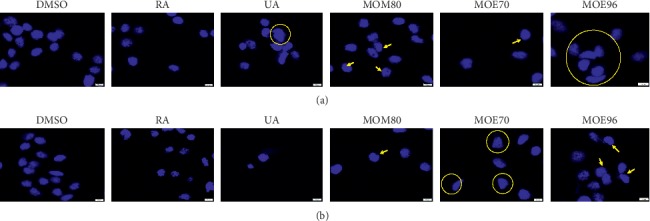
Morphological aspects of MCF-7 cells' nuclei stimulated with MO extracts: 50 *μ*g/mL (a) and 100 *μ*g/mL(b), standard compounds RA and UA: 50 *μ*M (a) and 100 *μ*M (b) and corresponding concentrations of DMSO, used as negative control, at 24h posttreatment. The scale bars represent 10 *μ*m.

**Figure 9:**
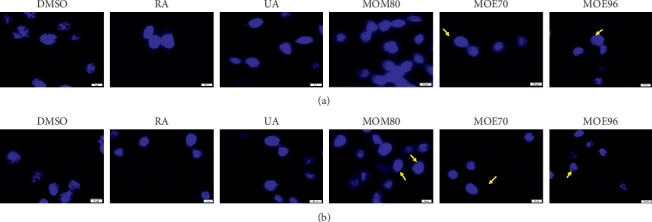
Morphological aspects of MDA-MB-231 cells' nuclei stimulated with MO extracts: 50 *μ*g/mL (a) and 100 *μ*g/mL(b), standard compounds RA and UA: 50 *μ*M (a) and 100 *μ*M (b) and corresponding concentrations of DMSO, used as negative control, at 24 h posttreatment. The scale bars represent 10 *μ*m.

To investigate if MO extracts and standard compounds (RA and UA) have the potential to induce apoptosis of breast adenocarcinoma MCF-7 and MDA-MB-231 cells, DAPI staining was employed. The results are presented in Figures 8 and 9 where the stained nuclei of MCF-7 cells and MDA-MB-231 cells, respectively, were capture on images and analyzed through fluorescent microscopy.

DAPI nuclear staining revealed different responses in terms of apoptosis after the treatment with the tested samples, dependent of the cell line genotype (ER−/ER+). Typical signs of apoptosis (chromatin condensation and nuclear membrane blebbing) [62] were marked with yellow arrows, while alterations of cell nuclei morphology were highlighted by a yellow circle, considered also as a specific feature of apoptosis [63].

MCF-7 cells incubated with MO extracts exhibited an increase of apoptotic markers as compared to the effect observed in the case of MDA-MB-231 cells. Among the MO extracts, the most significant apoptotic effects were induced by MOE96 followed by MOE70 on MCF-7 cell line, observing the loss of round-shape nucleus in several cells (surrounded by a yellow circle, Figure 8) together with chromatin condensation (marked with yellow arrows, Figure 8). However, MOM80 also promoted apoptotic specific signs—chromatin condensation and nuclear membrane blebbing on MCF-7 cells, data consistent with the results obtained by the cell viability assessment.

The ER− cancer cell line MDA-MB-231 seemed to be less affected by the exposure to MO extracts for 24 h, yet some apoptotic signs can be observed especially in the case of MOE70 and MOE96 (indicated by yellow arrows, Figure 9).

The treatment of both cell lines with DMSO and RA displayed equally dispersed chromatin density and round-shape nuclei, whereas UA induced cytopathic aspects specific of apoptosis—chromatin condensation and DNA fragmentation, along with morphological alterations of cells' nuclei.

Altogether, it is important to notice that the cytotoxic effect manifested by the MO extracts is achieved by apoptosis. In agreement with our study, Weidner et al. also reported that MO hydroethanolic extract at concentration of 600 *μ*g/mL induced a proapoptotic effect on HT-29 colon cells, after 48 h posttreatment by assessing caspase 3 and 7 cleavage, together with DAPI staining [64]. In addition, MO essential oil was also associated with apoptosis induction of human glioblastoma A242 and U87 cell lines, showing plasma membrane blebs, 48 hours posttreatment. Furthermore, the apoptosis process was endorsed by DNA fragmentation and caspase-3 activation [65].

The current investigation of MO extracts and pure standard compounds which are part of the extracts' composition revealed that the total MO extracts exert a significant chemopreventive activity on the two phenotypically different breast cancer cell lines by reducing the tumor angiogenesis process, influencing the tumor cell viability without affecting the healthy types of cells. A preventive effect against breast tumor cell invasiveness was registered for all tested samples. No areas of tumor cells outside the application ring were observed for any of the tested groups, pure compounds or multicompound lemon balm extracts.

Lemon balm extracts were evaluated here for the first time using the CAM assay, in order to analyze the potential alteration of the angiogenesis process in a breast cancer microenvironment. Our results indicated that the 96% ethanolic extract MOE96 exhibited a strong effect towards the limitation of breast cancer cell growth and tumor formation, also reducing the tumor angiogenic effect *in ovo*.

The effect of MOE96 was highly significant on limiting growth and tumor cell adherence in case of MCF7 cells 24 hours posttreatment, while the angiogenic process was slightly influenced 24 hours after inoculation for both tumor cell lines. When comparing the modifications induced after 24 hours of incubation, the antiangiogenic effect is observed only on the MCF-7 cells in terms of reduced number of complexes and by all samples except MOE70. However, 96 hours posttreatment, a more pronounced antiangiogenic effect was recorded in the case of MDA-MB-231 cells, in terms of reducing the number of tubular complexes, length, diameter, and branching pattern, compared to the effect observed after 24 hours. The most potent antiangiogenic effect was registered for the MOE96 extract on the MDA-MB-231 cell line, in correlation with the strongest effect of UA that exceeds RA in this particular type of extract (RA : UA, 1 : 2).

The pure compounds were less active against tumor formation and acted as antiangiogenic compounds especially 96 hours postinoculation with MDA-MB-231 cells. RA reduced all angiogenic measured parameters 96 hours posttreatment for the MDA-MB-231 cells. UA affected strongly the process of tumor angiogenesis, reducing the number of blood vessel complexes for both cancer types. Still, the total extracts, in particular MOE96, exhibited more important reduction of the angiogenesis process compared to RA on both cell lines after short and long stimulation, while reducing the size of the blood vessels comparable to the effect of 50 *μ*M UA, a much higher concentration than that present in the tested extracts.

The 96% ethanolic extract MOE96 induced the lowest cell viability of breast cancer cells *in vitro*, with a slightly stronger effect towards the estrogen receptor positive type MCF7 cell line, consistent with the proapoptotic assessment.

When compared to the effect of pure RA and UA, MOE96 exhibited a potent cytotoxic effect at a much lower concentration in active principles. MOE96, with a concentration ratio RA : UA of 1 : 2 induced a stronger inhibitory effect on both cell lines, in a dose-dependent manner, with a slightly higher potency against the MCF-7 cell line. MOE96 extract containing the equivalent of 1.34 μM antiproliferative UA and 0.97 μM of the proproliferative RA decreased breast cancer cell viability with about 30% in both cell lines, while a much higher UA concentration (25 μM) was needed in order to reduce tumor cell viability with approximately 50%.

Among the MO extracts, the most significant proapoptotic effect was induced by MOE96 followed by MOE70 on MCF-7 cell line, observing the loss of round-shape nucleus in several cells. The ER− cancer cell line MDA-MB-231 seemed to be less affected by exposure to MO extracts for 24 h, yet some apoptotic signs can be observed especially in the case of MOE70 and MOE96.

A multitargeted effect of the multiple components found in the composition of the MOE96 total extract together with a concentration ratio of RA : UA in favor of UA could be suggested as an explanation for the cytotoxic activity against breast cancer cells, but not affecting healthy cells. In addition, the study revealed that the reduction of breast cancer progression is not manifested through an antioxidant mechanism but rather through the proapoptotic activity and antiangiogenic effect of the extract with the concentration ratio RA : UA in favor of UA and presumably potentiated by the association with other polyphenols and pentacyclic triterpenes.

## 4. Conclusions

Various types of extracts from lemon balm (*Melissa officinalis* L.), a highly available and used medicinal plant in the Western area of Romania, were previously reported with promising antitumor effects. Our work contributed to this research topic by evaluating breast cancer antiangiogenic effect and chemoprevention induced by methanolic and ethanolic lemon balm leaves extracts. Although the highest contents in both polyphenols and triterpenes were detected in the 80% methanolic extract, with rosmarinic acid and ursolic acid as the most concentrated compounds, the highest cell inhibitory activity was exhibited by the 96% ethanolic extract, in particular against the estrogen receptor positive MCF7 breast cancer cell line, with no cytotoxic effect on healthy cells. The estrogen receptor positive MCF7 cell line proved to be more sensitive to the extract's antiproliferative activity than the triple negative MDA-MB-231 breast cancer cell line. The *in ovo* CAM assessment also revealed the 96% ethanolic extract as the most potent chemopreventive agent, inhibiting breast cancer progression and angiogenesis for the MDA-MB-231 breast cancer cell line. The antioxidant potential was directly correlated with the concentration of rosmarinic acid. Nevertheless, the chemopreventive potential of MOE96 extract is phenotype-dependent and is rather related to the apoptosis and antiangiogenic effects suggesting a multitargeted mechanism of action due to its multiple compound composition next to a concentration ratio of RA : UA in favor of UA.

## Figures and Tables

**Table 1:** Polyphenolic profile of analyzed samples: MOE70, MOE96, and MOM80 by LC-MS quantified in *μ*g/g d.w.

No.	Compound name	Rt (min)	[M-H^+^]^+^ (*m/z*)	MOE70 (*μ*g/g d.w.)	MOE96 (*μ*g/g d.w.)	MOM80 (*μ*g/g d.w.)
1	Caftaric acid	1.96	311	81.12	1.85	344.34
2	Gentisic acid	2.67	153	10.40	—	60.48
3	Chlorogenic acid	6.45	353	0.62	—	15.12
4	Caffeic acid	6.97	179	180.54	39.38	860.72
5	P-Coumaric acid	10.56	163	6.24	1.06	20.72
6	Ferulic acid	13.91	193	2.50	1.03	16.80
7	Sinapic acid	15.90	223	—	—	—
8	Hyperoside	21.56	463	4.78	3.30	16.24
9	Isoquercitrin	22.50	463	15.39	6.82	162.40
10	Rutin	23.01	609	8.11	135.74	847.28
11	Rosmarinic acid	24.05	359	8627.84	3515.60	86637.60
12	Myricetin	24.29	317	3.95	3.45	17.92
13	Fisetin	25.68	285	12.27	11.00	56.00
14	Quercitrin	26.18	447	2.50	2.86	6.16
15	Quercetol	30.38	301	8.11	5.72	33.60
16	Luteolin	32.78	285	15.18	—	26.32
17	Kaempferol	35.63	285	—	—	21.84
18	Apigenin	36.91	269	1.66	0.66	11.76

**Table 2:** Pentacyclic triterpenic compounds screened in analyzed samples by LC-MS quantified in *μ*g/g d.w.

No.	Compound name	Rt (min)	[M-H^+^]^+^ (*m/z*)	MOE70 (*μ*g/g d.w.)	MOE96 (*μ*g/g d.w.)	MOM80 (*μ*g/g d.w.)
1	Ursolic acid (UA)	50.96	455	3577.00	6103.47	11234.97
2	Oleanolic acid (OA)	57.50	455	915.03	1465.22	6151.67
3	Betulinic acid (BA)	61.70	455	12.85	16.61	169.88

## Data Availability

All data used to support the findings of this study are included within the article.
